# Phenotype-Associated Renal and Systemic Patterns During Short-Term Reduced-Tidal-Volume Mechanical Ventilation in Obese and Endotoxemic Rats: An Exploratory Study

**DOI:** 10.3390/metabo16090676

**Published:** 2026-09-14

**Authors:** Luciana Jorge, Bianca Castino Junqueira Silva, Marcia Bastos Convento, Clara Versolato Rasvisckas, Maria Aparecida da Glória, Cassiane Dezoti da Fonseca, Eloiza de Oliveira Silva, Maria de Fatima Fernandes Vattimo, Fernanda Teixeira Borges

**Affiliations:** 1 Nephrology Division, Department of Medicine, Federal University of São Paulo, São Paulo 04038-901, SP, Brazil; luciana.jorge@unifesp.br (L.J.); bcastino95@gmail.com (B.C.J.S.); claraversolato@gmail.com (C.V.R.); gloria@nefro.epm.br (M.A.d.G.); ft.borges@unifesp.br (F.T.B.); 2 Interdisciplinary Postgraduate Program in Health Sciences, Cruzeiro do Sul University, São Paulo 01506-000, SP, Brazil; 3 Paulista School of Nursing, Federal University of São Paulo, São Paulo 04024-002, SP, Brazil; cassiane.dezoti@unifesp.br; 4 School of Nursing, University of São Paulo, São Paulo 05403-000, SP, Brazil; eloizaosilva@usp.br (E.d.O.S.); nephron@usp.br (M.d.F.F.V.)

**Keywords:** mechanical ventilation, inflammation, acid–base balance, renal markers, lactate metabolism

## Abstract

**Background:** Mechanical ventilation (MV) may be associated with extrapulmonary responses shaped by the host inflammatory-metabolic background. **Methods:** This exploratory study characterized renal changes during 1 h of reduced-tidal-volume mechanical ventilation in male Wistar rats with diet-induced obesity or lipopolysaccharide (LPS)-induced endotoxemia, together with concurrent respiratory, acid–base, metabolic, and hemodynamic alterations. Arterial blood gas, biochemical, hemodynamic, and urinary variables were assessed at the pre-MV and post-MV time points. Renal protein expression was evaluated only at the post-MV time point. **Results:** The arterial partial pressure of carbon dioxide (PaCO_2_) decreased significantly in both groups. In the Obese group, metabolic acid–base abnormalities persisted and were accompanied by increases in serum urea and the urinary protein-to-creatinine ratio. In the LPS group, lactate increased, bicarbonate decreased, and mean arterial pressure and serum creatinine increased. At the post-MV time point, renal interleukin-6 expression was lower in the LPS group than in the Obese group, whereas renal tumor necrosis factor-alpha expression was higher in the LPS group than in the Obese group. **Conclusions:** These results support interpreting the physiological and renal changes observed in association with the integrated experimental protocol, including mechanical ventilation, in relation to the host’s underlying pathophysiology and inflammatory-metabolic context.

## 1. Introduction

Mechanical ventilation (MV) is an essential intervention in the management of acute respiratory failure, but its effects extend beyond the pulmonary compartment. Positive-pressure ventilation may influence systemic hemodynamics, acid–base balance, metabolism, inflammatory responses, and renal function through changes in intrathoracic pressure, venous return, cardiac output, renal blood flow, and glomerular filtration [1]. In addition, MV may contribute to alterations in renal function through inflammatory lung–kidney crosstalk [2].

Despite the adoption of protective ventilatory strategies, MV remains associated with a risk of acute kidney injury, and distinguishing the effects of ventilatory support from those related to the underlying disease remains challenging in critically ill patients [3,4]. Because the response to ventilation is influenced by respiratory mechanics, underlying pathophysiology, and host comorbidities [5,6], distinct inflammatory microenvironments may modulate systemic responses during reduced-tidal-volume ventilation, particularly with respect to acid–base balance, lactate metabolism, hemodynamics, and renal function.

Obesity represents a state of chronic low-grade metabolic inflammation [7] and is associated with reduced functional residual capacity, increased pleural pressure, decreased respiratory system compliance, atelectasis, hypoxemia, and greater susceptibility to hypercapnia [8]. Although obesity is associated with an increased need for ventilatory support, some studies have reported mortality rates similar to those observed in patients without obesity, a phenomenon referred to as the “obesity paradox” [9,10]. However, most studies of MV in obesity have focused predominantly on pulmonary variables [11]. In contrast, metabolic and renal responses remain less well characterized despite the systemic and renal vulnerability associated with this phenotype [12].

In contrast, lipopolysaccharide (LPS)-induced endotoxemia represents a model of acute systemic inflammation characterized by marked immune activation, cytokine release, increased vascular permeability, pulmonary impairment, hemodynamic alterations, and metabolic dysfunction [13]. During mechanical ventilation, this context is particularly relevant because pulmonary inflammation, circulatory instability, lactate accumulation, and renal vulnerability may coexist, thereby complicating the attribution of systemic responses exclusively to ventilation [14].

Thus, obesity and endotoxemia represent distinct inflammatory microenvironments: chronic low-grade metabolic inflammation in obesity and acute endotoxin-driven inflammation in endotoxemia. Distinguishing ventilation-related biotrauma from the pre-existing inflammatory state therefore remains an important analytical challenge [15]. This complexity is consistent with the “two-hit model,” in which an initial insult may sensitize pulmonary, vascular, and renal tissues before mechanical ventilation is superimposed on an already inflamed metabolic background [16,17,18]. Accordingly, molecular, metabolic, and renal alterations should not be attributed exclusively to ventilatory support in the presence of pre-existing inflammation.

In this context, diet-induced obesity and LPS-induced endotoxemia represent contrasting inflammatory microenvironments: the former characterized by chronic, low-grade metabolic inflammation, and the latter by acute endotoxin-driven inflammation. Given their distinct baseline pathophysiology, the primary analytical approach focused on pre-to-post changes within each phenotype rather than on direct comparisons of change magnitudes between phenotypes. This approach allowed each group to be interpreted relative to its own baseline state. Ventilatory settings were tailored to each experimental condition within a reduced-tidal-volume strategy intended to provide protective ventilation.

Accordingly, the primary outcome domain of this exploratory study consisted of pre-to-post changes in serum and urinary renal biochemical parameters during 1 h of reduced-tidal-volume mechanical ventilation in male Wistar rats with diet-induced obesity or LPS-induced endotoxemia. Concurrent respiratory, acid–base, metabolic, and hemodynamic alterations were evaluated as complementary exploratory outcomes. Because molecular analyses were performed only at the post-MV time point, direct between-phenotype comparisons were restricted to renal protein expression of interleukin-10 (IL-10), interleukin-6 (IL-6), tumor necrosis factor-alpha (TNF-α), and angiotensin II type 1 receptor (AT1R), and were interpreted as phenotype-associated molecular patterns rather than as evidence of temporal mechanisms.

## 2. Materials and Methods

### 2.1. Experimental Group Allocation and Mechanical Ventilation

All animal handling, housing, and experimental procedures were conducted in accordance with established institutional guidelines [19]. The study was approved by the Institutional Animal Care and Use Committee under protocol CEUA 2994070823. Male Wistar rats aged 6–8 weeks, with an initial body weight of 190–210 g, were used. Animals were housed at the animal facility of the Federal University of São Paulo (UNIFESP), São Paulo, SP, Brazil, as needed for metabolic cage housing.

A total of 19 animals were included in the study and maintained in individual cages or housed in pairs, with ad libitum access to water and either standard or high-calorie chow. Ambient temperature was maintained between 22 and 26 °C, and animals were kept under a 12 h light/dark cycle. Following a 7-day acclimatization period, the animals were assigned to predefined experimental protocols according to the planned phenotype-induction strategy. The Obese group underwent 90 days of high-fat/high-fructose feeding, whereas the LPS group was maintained on standard chow for the same 90-day period. On day 91, the Obese group underwent mechanical ventilation, while the LPS group received LPS and underwent mechanical ventilation 24 h later. Thus, animals in both groups were approximately 19–21 weeks old at the time of mechanical ventilation.

In the Obese group (*n* = 11), obesity was induced over 90 days using a high-fat/high-fructose diet with a caloric profile of 15.3% protein, 16.4% carbohydrates, and 68.3% fat. The diet formulation contained 20% fructose and 35% lard, with an energy density of 5.6 kcal/g (RH19543; Rhoster Indústria e Comércio Ltda., Araçoiaba da Serra, SP, Brazil). This protocol was based on a high-fat/high-fructose diet-induced obesity model previously standardized by our group to investigate obesity-associated renal, metabolic, hemodynamic, and structural alterations [7]. On day 91, the animals underwent mechanical ventilation.

In the LPS group (*n* = 8), endotoxemia was induced by intraperitoneal administration of lipopolysaccharide (LPS) from Escherichia coli O111:B4 (Sigma-Aldrich, St. Louis, MO, USA). LPS was administered at a dose of 5 mg/kg after dilution in phosphate-buffered saline (PBS) [20]. These animals received a standard diet containing 20.6% protein, 61.7% carbohydrates, 17.7% lipids, and 10% sucrose, with an energy density of 3.9 kcal/g (AIN-93; Rhoster Indústria e Comércio Ltda., SP, Brazil). Twenty-four hours after LPS administration, the animals underwent mechanical ventilation.

Before surgical preparation, animals received morphine (2.5 mg/kg, intraperitoneally) for analgesia, followed by sodium pentobarbital (50 mg/kg, intraperitoneally; Syntec, Barueri, SP, Brazil) for anesthesia. Supplemental pentobarbital was administered as needed to maintain an adequate anesthetic plane. During anesthetic induction and surgical preparation, animals received 100% oxygen. After tracheostomy, they were connected to a VentElite 55-7040 ventilator (Harvard Apparatus, Shanghai, China), designed for small animals and capable of operating in volume- or pressure-controlled modes. The device allows adjustment of tidal volume from 50 µL to 5 mL and peak inspiratory pressure from 0 to 50 cm of water (cmH_2_O).

Ventilatory settings were defined according to the experimental condition and documented throughout the protocol. The recorded variables included set tidal volume, applied positive end-expiratory pressure (PEEP), peak inspiratory pressure, respiratory rate, and fraction of inspired oxygen (FiO_2_). After connection to the ventilator, FiO_2_ was adjusted to 0.50.

The pre-MV arterial blood sample was collected immediately after connection to the ventilator, with FiO_2_ set at 0.50, and before the start of the predefined 1 h experimental ventilation period. No separate pre-protocol ventilation period was applied. FiO_2_ was maintained at 0.50 throughout the subsequent ventilation period. At the end of the 1 h experimental ventilation period, the post-MV arterial blood sample was collected. During the pre-MV period, rats were housed individually in metabolic cages for 24 h with ad libitum access to water and standard chow for urine collection. Urine was collected under a layer of liquid paraffin; total urinary volume was measured, and the paraffin layer was subsequently removed using a Pasteur pipette. Samples were centrifuged, aliquoted into microtubes, and stored at −20 °C. At the post-MV time point, urine was collected directly from the urinary bladder at the end of the experimental protocol. Rats were euthanized at the end of the experimental protocol by intraperitoneal administration of a lethal dose of xylazine (30 mg/kg) and ketamine (270 mg/kg).

In the Obese group, animals were mechanically ventilated for 1 h using a reduced-tidal-volume strategy adapted to the respiratory mechanics associated with obesity and intended to limit ventilation-associated lung injury. The recorded set tidal volume ranged from 2.5 to 4.0 mL and, after normalization to body weight, corresponded to 5.90 ± 0.62 mL/kg. Peak inspiratory pressure was 17.8 ± 2.1 cmH_2_O, PEEP was maintained at 1 cmH_2_O, respiratory rate at 90 breaths/min, and FiO_2_ at 0.50. The use of reduced tidal volumes was based on protective ventilation principles described in obesity; whereby tidal volume limitation is intended to reduce excessive alveolar distension. The interpretation of airway pressure in obesity also considered the contribution of increased chest wall and pleural pressures to measured airway pressure [21,22,23,24]. A low PEEP level was selected to avoid zero PEEP while limiting the potential hemodynamic effects of higher positive-pressure levels during the short-term protocol [23,24].

**Figure 1:**
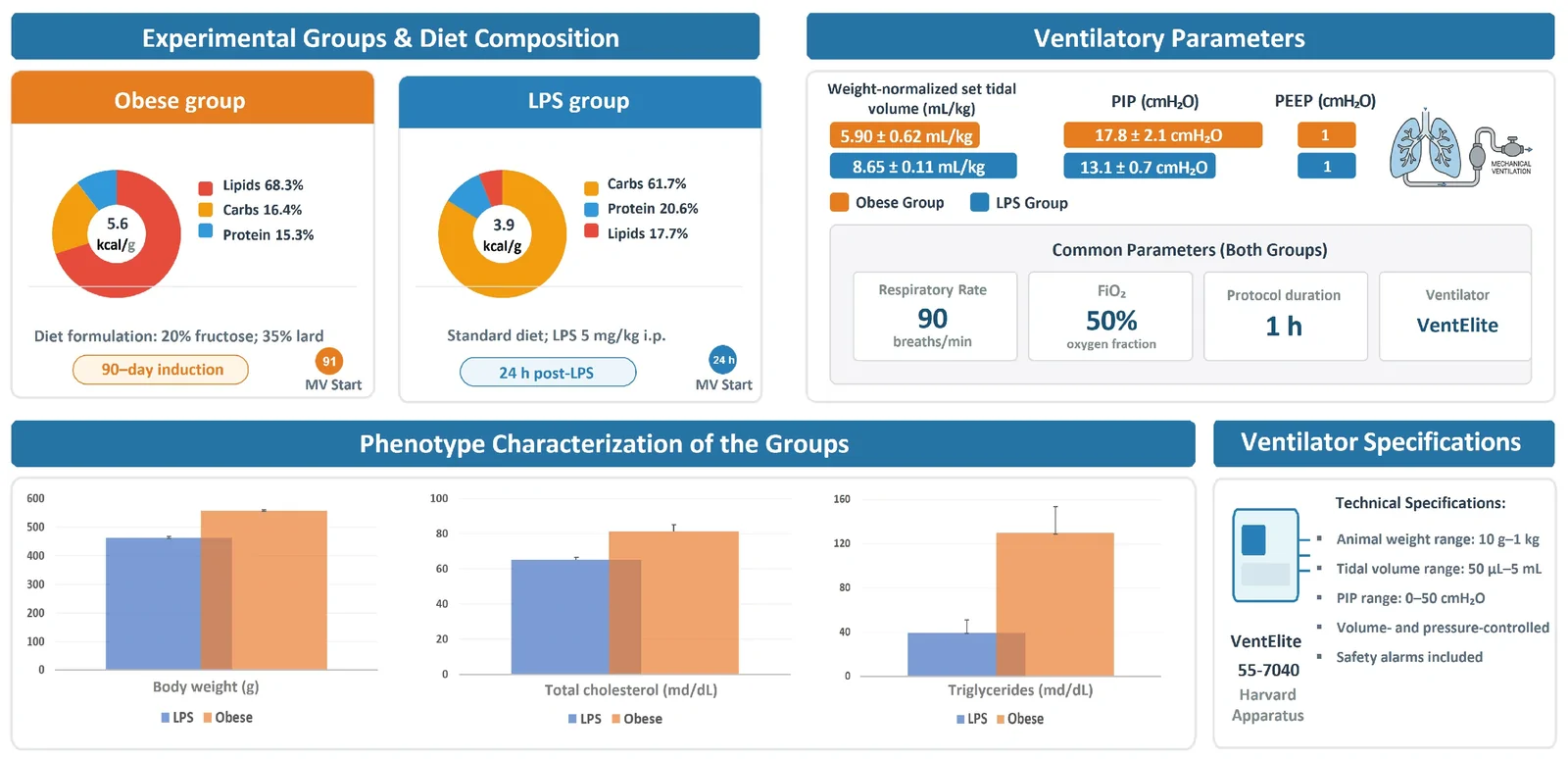
Experimental design, ventilatory settings, and phenotype characterization. Values are presented as mean ± SEM. Final body weight was 557.13 ± 10.14 g in the Obese group and 462.75 ± 4.33 g in the LPS group. Total cholesterol was 81.17 ± 8.51 mg/dL in the Obese group and 65.00 ± 1.54 mg/dL in the LPS group. Triglyceride levels were 129.83 ± 23.61 mg/dL and 39.67 ± 11.68 mg/dL, respectively.

In the LPS group, animals were mechanically ventilated for 1 h using a ventilation strategy intended to limit ventilation-associated lung injury, based on experimental studies of LPS-induced lung injury and protective ventilation principles applied in acute respiratory distress syndrome [14,25]. The ventilator was set to deliver a nominal tidal volume of 4.0 mL, corresponding to 8.65 ± 0.11 mL/kg after normalization to body weight (*n* = 8). Peak inspiratory pressure was 13.1 ± 0.7 cmH_2_O, PEEP was maintained at 1 cmH_2_O, respiratory rate at 90 breaths/min, and FiO_2_ at 0.50. These settings were selected to limit exposure to tidal volumes associated with experimental ventilator-induced lung injury. A PEEP of 1 cmH_2_O was maintained as a conservative approach, avoiding zero PEEP while limiting the potential hemodynamic effects of higher levels of positive end-expiratory pressure during the short-term protocol [26,27]. Figure 1 summarizes the experimental design.

### 2.2. Arterial Blood Gas Analysis and Acid–Base Parameters

Arterial blood gas analysis was performed during the pre-MV and post-MV periods. The following parameters were assessed: pH, partial pressure of carbon dioxide (PaCO_2_), partial pressure of oxygen (PaO_2_), plasma bicarbonate (HCO_3_^−^), chloride (Cl^−^), hematocrit (Ht), lactate, sodium (Na^+^), potassium (K^+^), ionized calcium (Ca^2+^), blood base excess (BE-B), and non-fasting blood glucose. Analyses were performed using a previously calibrated GEM Premier 3500 analyzer with an iQM 150 GEM Premier cartridge (Instrumentation Laboratory Co., Bedford, MA, USA), according to the manufacturer’s instructions. Hemoglobin (Hb) was estimated from hematocrit using the following formula: Hb (g/dL) = Ht (%)/3. The PaO_2_/FiO_2_ ratio was calculated with FiO_2_ expressed as a decimal fraction and used as an index of oxygenation and pulmonary gas exchange [28].

### 2.3. Acid–Base Indices

The acid–base indices were calculated during the pre-MV and post-MV periods using the following formulas [29]: anion gap = Na^+^ − (Cl^−^ + HCO_3_^−^), where Na^+^ represents serum sodium concentration, Cl^−^ represents serum chloride concentration, and HCO_3_^−^ represents plasma bicarbonate concentration. The apparent strong ion difference (SID) was calculated as SID = (Na^+^ + K^+^) − Cl^−^, where K^+^ represents serum potassium concentration [30].

### 2.4. Assessment of Alveolar Oxygen Pressure and the Alveolar–Arterial Oxygen Gradient

Alveolar oxygen pressure (PAO_2_) was estimated for each animal at the pre-MV and post-MV time points using the alveolar gas equation: PAO_2_ = FiO_2_ × (PB − PH_2_O) − PaCO_2_/RQ, assuming FiO_2_ = 0.50, barometric pressure (PB) = 760 mmHg, water vapor pressure (PH_2_O) = 47 mmHg, and a respiratory quotient (RQ) of 0.8. The corresponding alveolar–arterial oxygen gradient was then calculated as the A–a gradient = PAO_2_ − PaO_2_ [31].

### 2.5. Assessment of Mean Arterial Pressure (MAP)

Mean arterial pressure (MAP) was assessed invasively during the pre-MV and post-MV periods. After confirmation of an adequate anesthetic plane, the animals were placed in the supine position, and the ventral cervical region was shaved. The left carotid artery was exposed through a cervical approach, with careful identification of the neurovascular bundle to minimize vascular injury and vagal stimulation. The artery was then catheterized with PE-50 polyethylene tubing for arterial blood sampling and continuous hemodynamic monitoring. MAP was recorded using a BIOPAC data acquisition system (BIOPAC Systems Inc., Goleta, CA, USA). After catheterization, the animals underwent tracheostomy for insertion of a tracheal cannula and connection to the mechanical ventilator.

### 2.6. Biochemical Assessment of Renal Parameters

Renal parameters were assessed during the pre-MV and post-MV periods by measuring serum and urinary urea and creatinine concentrations, and proteinuria. Urea concentrations were determined using a commercial enzymatic-colorimetric kit (Labtest Diagnóstica SA., Lagoa Santa, MG, Brazil), according to the manufacturer’s instructions. The method was based on urease-mediated hydrolysis of urea, followed by the formation of indophenol blue, with absorbance measured at 620 nm. Serum and urinary creatinine concentrations were determined using the modified Jaffe method [32]. For creatinine measurement, serum and urine samples were homogenized, incubated for 10 min, centrifuged for 10 min, incubated again for 15 min at 25 °C, and analyzed using a Multiskan EX microplate reader (Labsystems, Vantaa, Finland). Proteinuria was measured using a commercial enzymatic-colorimetric kit (Labtest Diagnóstica SA., Lagoa Santa, MG, Brazil) and normalized to urinary creatinine.

### 2.7. Western Blotting

Protein expression was assessed by Western blotting in kidney tissues collected during the post-MV period. Proteins were extracted using 600 µL of radioimmunoprecipitation assay (RIPA) buffer supplemented with protease inhibitors, and protein concentration was determined using the Lowry method [33] with a bovine serum albumin standard curve. Thirty micrograms of total protein were loaded per lane, mixed with denaturing sample buffer, heated at 95 °C for 5 min, and subsequently cooled on ice. Proteins were separated by SDS-polyacrylamide gel electrophoresis using approximately 10% resolving gels and 4% stacking gels. Electrophoresis was initially performed at 60 V and subsequently at 120 V for approximately 90 min. Proteins were transferred to nitrocellulose membranes using a Trans-Blot^®^ Turbo™ Transfer System (Bio-Rad Laboratories, Hercules, CA, USA). Membranes were blocked with 5% bovine serum albumin in TBS-T for 1 h at room temperature, washed three times for 10 min in TBS-T, and incubated overnight with primary antibodies against interleukin-10 (IL-10; rat monoclonal antibody; catalog no. MA5-23796; 1:1000; Thermo Fisher Scientific, Waltham, MA, USA), interleukin-6 (IL-6; rabbit polyclonal IgG; catalog no. NB600-1131; 1:1000; Novus Biologicals, Centennial, CO, USA), tumor necrosis factor-alpha (TNF-α; rabbit antibody; catalog no. ab66579; 1:1000; Abcam, Waltham, MA, USA), angiotensin II type 1 receptor (AT1R/AGTR1; rabbit monoclonal antibody [EPR3873]; catalog no. ab124734; 1:1000; Abcam, Waltham, MA, USA), and beta-actin (β-actin; rabbit antibody; catalog no. ab8227; 1:1000; Abcam, Waltham, MA, USA). After washing three times with TBS-T, the membranes were incubated for 1 h at 4 °C with the corresponding HRP-conjugated secondary antibodies (1:30,000; Cell Signaling Technology, Danvers, MA, EUA). Chemiluminescent detection was performed using ECL Plus, and the bands were visualized with an Amersham™ Imager 600 system (GE Healthcare, Chicago, IL, USA). Densitometric analysis was performed using ImageJ software (version 1.53k; National Institutes of Health, Bethesda, MD, USA), and the results were expressed as the ratio of the protein of interest to β-actin. Western blot analyses included three biological replicates per phenotype (Obese, *n* = 3; LPS, *n* = 3), selected based on the availability of kidney tissue with sufficient quantity and quality to allow loading of at least 30 µg of total protein per lane for Western blot analysis. Sample selection was completed before densitometric quantification and statistical analysis.

### 2.8. Statistical Analysis

Statistical analyses were performed using jamovi, version 2.6, for Windows. For within-phenotype comparisons between the pre-MV and post-MV time points, the normality assumption was assessed on the distribution of the paired differences (Δ = post-MV − pre-MV) using the Shapiro–Wilk test. Paired Student’s *t* tests were used when the paired differences were normally distributed; otherwise, Wilcoxon signed-rank tests were applied. Given the small effective sample sizes for some outcomes, formal normality testing was interpreted with caution. For statistically significant parametric paired comparisons, mean paired differences, 95% confidence intervals (CIs), exact *p*-values, and effect sizes (Cohen’s d) with 95% CIs were reported.

For Western blot analyses performed at the post-MV time point, comparisons between the Obese and LPS groups were conducted using independent-samples Student’s *t* tests when data were normally distributed; otherwise, Mann–Whitney U tests were applied. For statistically significant parametric between-group comparisons, mean differences, 95% CIs, exact *p*-values, and effect sizes (Cohen’s d) with 95% CIs were reported.

Given the small sample size and exploratory nature of the study, no adjustment for multiplicity across outcomes was applied. Accordingly, all reported *p*-values are unadjusted and should be interpreted as exploratory and hypothesis-generating. Results are presented as mean ± standard error of the mean (SEM) and/or mean ± standard deviation (SD), as indicated in the corresponding tables and figure legends. A two-sided *p*-value ≤ 0.05 was considered statistically significant. This approach was retained to preserve the predefined exploratory analytical framework and avoid overinterpretation of individual statistical findings overall.

## 3. Results

### 3.1. Ventilatory Response and Metabolic Acid–Base Profile

Table 1 summarizes the arterial blood gas and hemodynamic parameters in the Obese and LPS groups before and after mechanical ventilation.

In the Obese group, arterial pH increased significantly from 7.1 ± 0.06 at pre-MV to 7.2 ± 0.02 at post-MV, corresponding to a mean paired increase of 0.148 (95% CI, 0.008 to 0.287), t(7) = 2.50, *p* = 0.041, Cohen’s d = 0.88 (95% CI, 0.03 to 1.69). PaCO_2_ also decreased significantly, from 51.9 ± 6.3 mmHg at pre-MV to 31.3 ± 2.1 mmHg at post-MV, with a mean paired decrease of 20.6 mmHg (95% CI, 7.38 to 33.9), t(5) = 4.00, *p* = 0.010, Cohen’s d = 1.63 (95% CI, 0.34 to 2.87).

PaO_2_ changed from 110.6 ± 36.2 to 132.2 ± 33.5 mmHg (*p* = 0.67), HCO_3_^−^ from 16.3 ± 1.6 to 14.1 ± 1.2 mEq/L (*p* = 0.33), chloride from 116.8 ± 1.9 to 117.2 ± 2.1 mmol/L (*p* = 0.88), BE-B from −10.8 ± 4.1 to −11.3 ± 3.1 mmol/L (*p* = 0.92), lactate from 2.7 ± 1.0 to 3.2 ± 0.6 mmol/L (*p* = 0.71), and MAP from 97.0 ± 9.9 to 102.2 ± 9.6 mmHg (*p* = 0.70). None of these pre-MV to post-MV changes were statistically significant.

In the LPS group, PaCO_2_ decreased significantly from 46.6 ± 10.3 mmHg at pre-MV to 21.4 ± 3.5 mmHg at post-MV, corresponding to a mean paired decrease of 25.1 mmHg (95% CI, 4.23 to 46.0), t(5) = 3.09, *p* = 0.027, Cohen’s d = 1.26 (95% CI, 0.13 to 2.34). HCO_3_^−^ also decreased significantly, from 19.6 ± 0.7 mEq/L at pre-MV to 12.0 ± 1.9 mEq/L at post-MV, with a mean paired decrease of 7.58 mEq/L (95% CI, 2.69 to 12.5), t(4) = 4.31, *p* = 0.013, Cohen’s d = 1.93 (95% CI, 0.35 to 3.45).

MAP increased significantly from 119.5 ± 6.3 mmHg at pre-MV to 148.5 ± 11.4 mmHg at post-MV, with a mean paired increase of 29.0 mmHg (95% CI, 8.96 to 49.0), t(3) = 4.60, *p* = 0.019, Cohen’s d = 2.30 (95% CI, 0.29 to 4.28). Lactate also increased significantly, from 1.5 ± 0.2 mmol/L at pre-MV to 4.8 ± 1.2 mmol/L at post-MV, corresponding to a mean paired increase of 3.33 mmol/L (95% CI, 0.11 to 6.56), t(5) = 2.66, *p* = 0.045, Cohen’s d = 1.09 (95% CI, 0.02 to 2.09).

By contrast, pH changed from 7.1 ± 0.1 to 7.2 ± 0.08 (*p* = 0.51), PaO_2_ from 80.2 ± 14.4 to 90.9 ± 14.6 mmHg (*p* = 0.62), chloride from 116.0 ± 3.2 to 113.0 ± 1.3 mmol/L (*p* = 0.43), and BE-B from −8.9 ± 7.1 to −5.6 ± 5.4 mmol/L (*p* = 0.72), with none of these pre-MV to post-MV changes reaching statistical significance.

**Figure 2:**
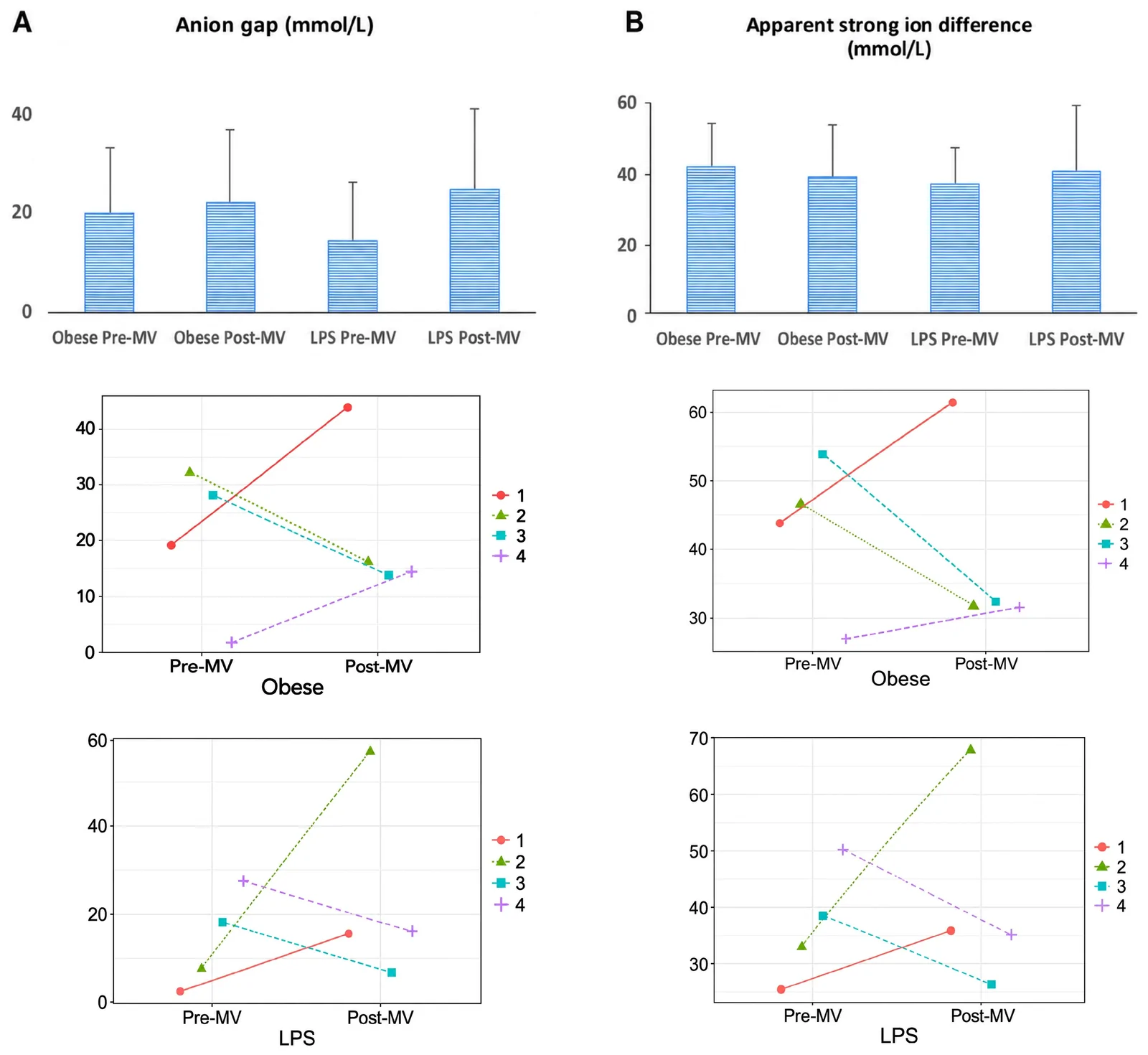
Acid–base indices at the pre-MV and post-MV time points of the reduced-tidal-volume mechanical ventilation protocol in Obese and LPS rats. (**A**) Anion gap (mmol/L). (**B**) Apparent strong ion difference (SID; mmol/L). The upper panel shows group means ± SD, whereas the lower panels show individual paired observations, with lines connecting pre-MV and post-MV values for each animal. Within-phenotype comparisons were performed using complete paired observations (Obese, *n* = 4; LPS, *n* = 4); no direct between-phenotype comparisons were conducted.

Figure 2 presents the anion gap (Figure 2A) and apparent strong ion difference (SID; Figure 2B) in the Obese and LPS groups at the pre-MV and post-MV time points. In the Obese group, the mean anion gap changed from 19.8 ± 13.9 mmol/L at pre-MV to 22.1 ± 14.6 mmol/L at post-MV (*p* = 0.83), whereas the mean SID changed from 42.2 ± 12.3 mmol/L to 39.3 ± 14.8 mmol/L (*p* = 0.77). These findings occurred in the context of lower HCO_3_^−^, a modest increase in lactate, stable sodium concentrations, and minimal variation in chloride. In the LPS group, the mean anion gap changed from 13.9 ± 11.3 mmol/L at pre-MV to 24.1 ± 22.7 mmol/L at post-MV (*p* = 0.45), while the mean SID changed from 36.8 ± 10.4 mmol/L to 41.1 ± 18.4 mmol/L (*p* = 0.70). These findings occurred alongside a significant reduction in HCO_3_^−^ and a significant increase in lactate. Sodium remained stable, whereas chloride decreased modestly post-MV.

### 3.2. Oxygenation Profile

**Figure 3:**
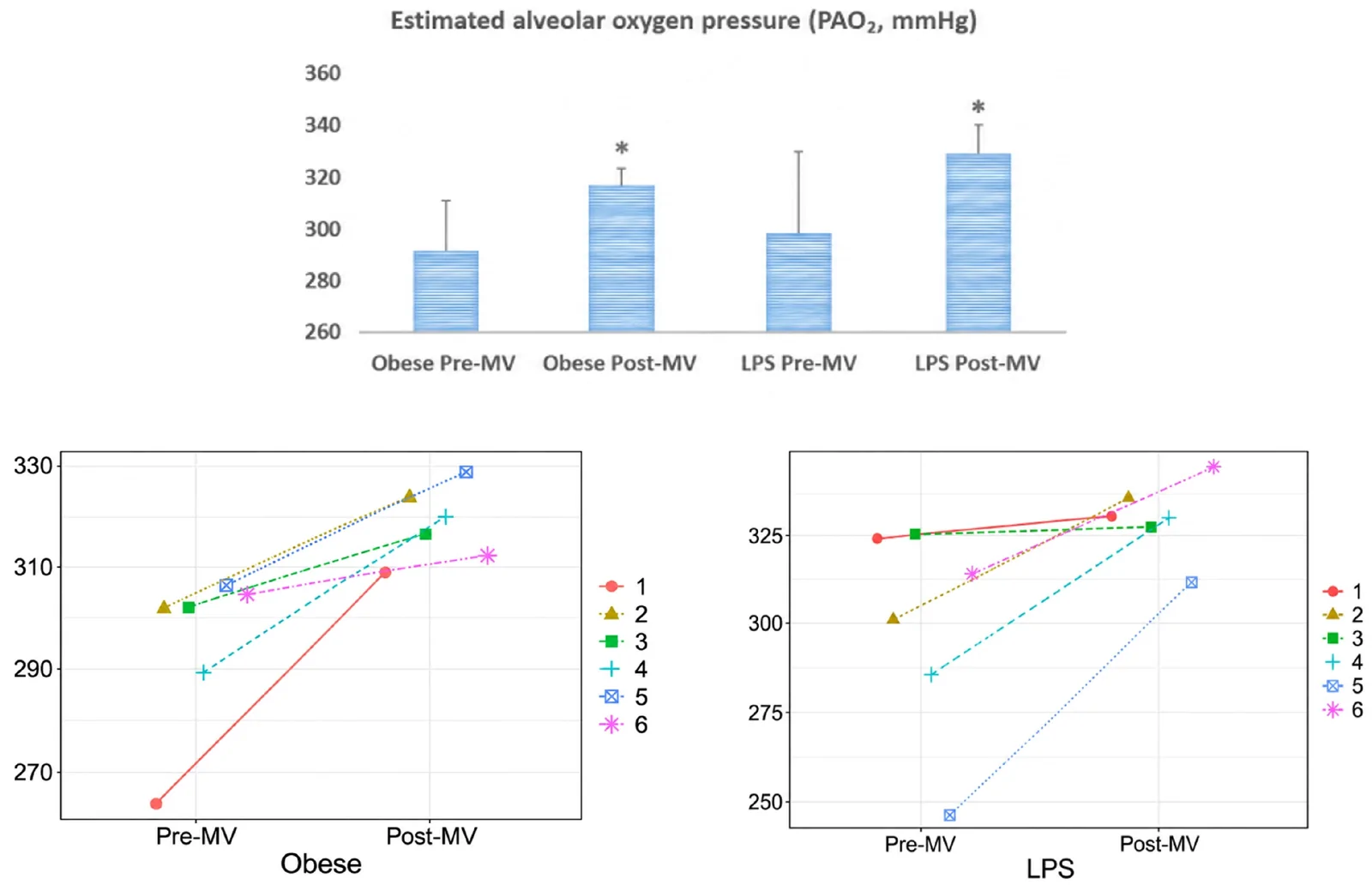
Estimated alveolar oxygen pressure (PAO_2_) at the pre-MV and post-MV time points of the reduced-tidal-volume mechanical ventilation protocol in Obese and LPS rats. The upper panel shows group means ± SD, whereas the lower panels show individual paired observations, with lines connecting pre-MV and post-MV values for each animal. Because PAO_2_ was mathematically derived from PaCO_2_ under fixed FiO_2_, barometric pressure, water-vapor pressure, and respiratory quotient assumptions, it was presented descriptively and was not interpreted as an independent inferential outcome. Analyses were based on complete paired observations (Obese, *n* = 6; LPS, *n* = 6); no direct between-phenotype comparisons were conducted. (*) *p* ≤ 0.05 was considered statistically significant for post-MV versus pre-MV comparisons within each phenotype.

Figure 3 shows the estimated alveolar oxygen pressure (PAO_2_) at the pre-MV and post-MV time points in the Obese and LPS groups. In the Obese group, estimated PAO_2_ increased significantly from 291.6 ± 19.4 mmHg at pre-MV to 317.4 ± 6.8 mmHg at post-MV, corresponding to a mean paired increase of 25.8 mmHg (95% CI, 9.22 to 42.4), *t*(5) = 4.00, *p* = 0.010, Cohen’s *d* = 1.63 (95% CI, 0.34 to 2.87). In the LPS group, estimated PAO_2_ also increased significantly from 298.2 ± 31.7 mmHg at pre-MV to 329.7 ± 10.9 mmHg at post-MV, corresponding to a mean paired increase of 31.4 mmHg (95% CI, 5.29 to 57.5), *t*(5) = 3.09, *p* = 0.027, Cohen’s *d* = 1.26 (95% CI, 0.13 to 2.34). Because estimated PAO_2_ was mathematically derived from PaCO_2_ under fixed FiO_2_, barometric pressure, water-vapor pressure, and respiratory quotient assumptions, it was not interpreted as an independent inferential outcome.

**Figure 4:**
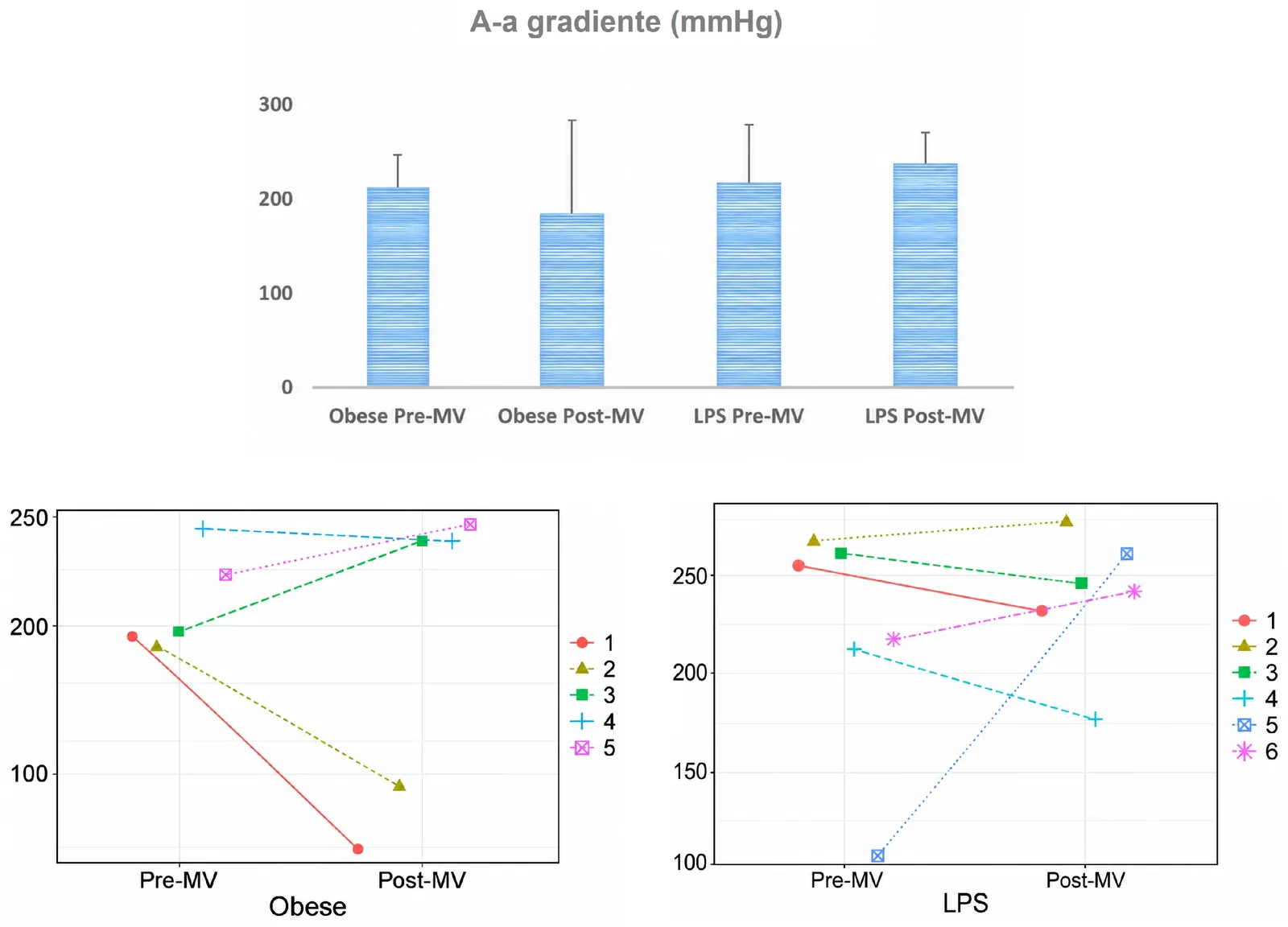
Alveolar–arterial oxygen gradient (A–a gradient) at the pre-MV and post-MV time points of the reduced-tidal-volume mechanical ventilation protocol in Obese and LPS rats. The upper panel shows group means ± SD, whereas the lower panels show individual paired observations, with lines connecting pre-MV and post-MV values for each animal. Within-phenotype comparisons were performed using complete paired observations (Obese, *n* = 5; LPS, *n* = 6); no direct between-phenotype comparisons were conducted.

Figure 4 shows the alveolar–arterial oxygen gradient at the pre-MV and post-MV time points in the Obese and LPS groups. In the Obese group, the mean A–a gradient changed from 212.9 ± 34.0 mmHg at pre-MV to 182.7 ± 106.2 mmHg at post-MV (*p* = 0.53). In the LPS group, the mean A–a gradient changed from 218.0 ± 61.3 mmHg at pre-MV to 238.8 ± 33.2 mmHg at post-MV (*p* = 0.48).

**Figure 5:**
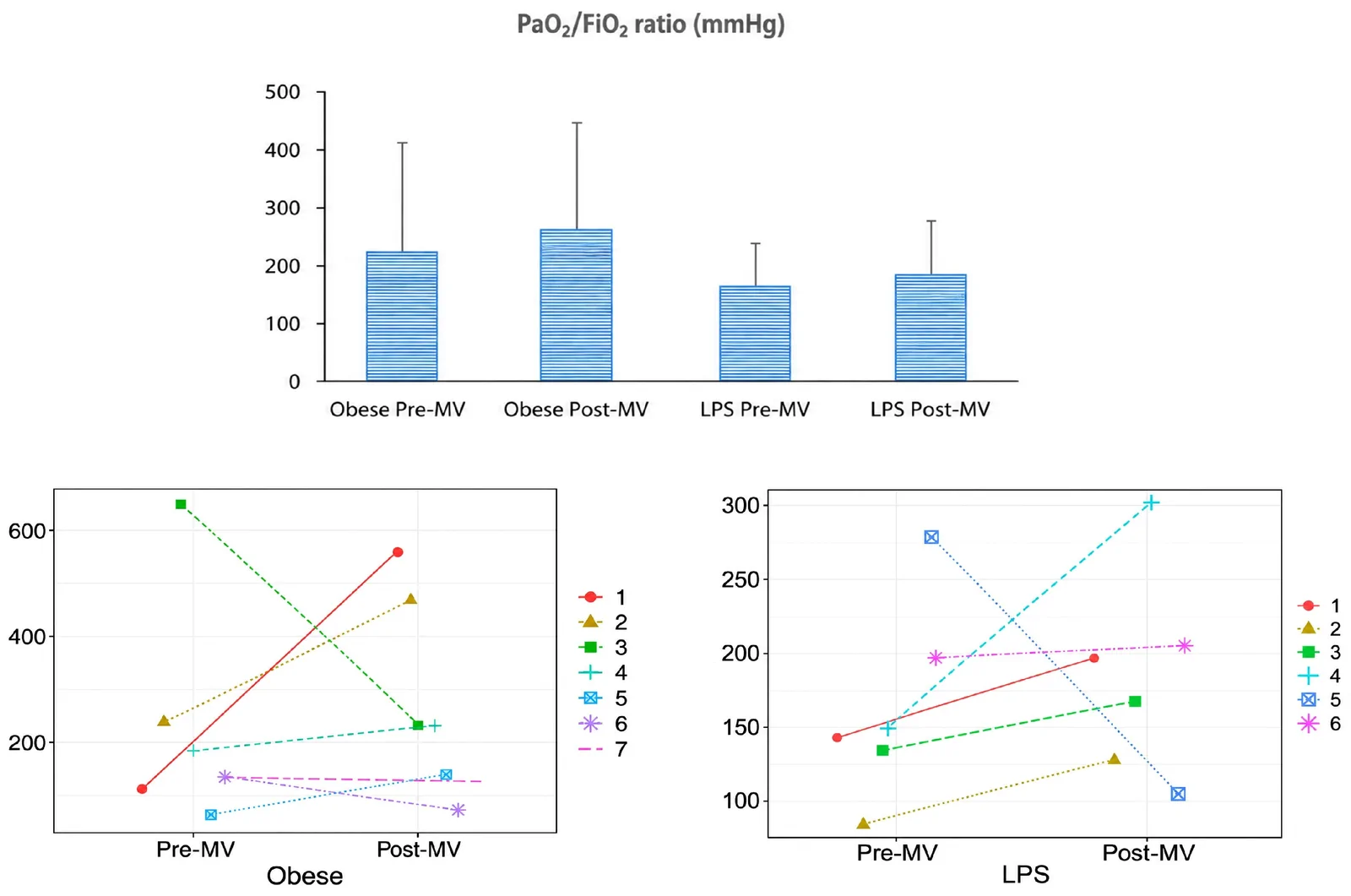
PaO_2_/FiO_2_ ratio at the pre-MV and post-MV time points of the reduced-tidal-volume mechanical ventilation protocol in Obese and LPS rats. The upper panel shows group means ± SD, whereas the lower panels show individual paired observations, with lines connecting pre-MV and post-MV values for each animal. Within-phenotype comparisons were performed using complete paired observations (Obese, *n* = 7; LPS, *n* = 6); no direct between-phenotype comparisons were conducted.

Figure 5 shows the PaO_2_/FiO_2_ ratio at the pre-MV and post-MV time points in the Obese and LPS groups. In the Obese group, the mean PaO_2_/FiO_2_ ratio changed from 221.3 ± 191.6 mmHg at pre-MV to 264.6 ± 177.4 mmHg at post-MV (*p* = 0.67). In the LPS group, the mean ratio changed from 160.4 ± 70.8 mmHg at pre-MV to 181.8 ± 71.9 mmHg at post-MV (*p* = 0.62).

### 3.3. Biochemical and Renal Parameters

Table 2 presents the biochemical parameters in the Obese and LPS groups at the pre-MV and post-MV time points. In the Obese group, sodium changed from 152.0 ± 2.7 to 153.5 ± 3.1 mEq/L (*p* = 0.72), potassium from 4.2 ± 0.2 to 4.2 ± 0.2 mEq/L (*p* = 0.81), ionized calcium from 0.5 ± 0.05 to 0.4 ± 0.06 mmol/L (*p* = 0.29), and non-fasting glucose from 276.0 ± 30.7 to 325.4 ± 32.4 mg/dL (*p* = 0.30). Hematocrit changed from 35.4 ± 1.8% to 34.4 ± 2.0% (*p* = 0.74), whereas estimated hemoglobin, calculated from hematocrit using the formula Hb (g/dL) = Ht (%)/3, changed from 11.8 ± 0.6 to 11.5 ± 0.7 g/dL. None of these pre-MV to post-MV changes reached statistical significance.

In the LPS group, sodium remained unchanged at 145.7 ± 3.1 mEq/L at pre-MV and 145.7 ± 5.5 mEq/L at post-MV (*p* = 1.00). Potassium changed from 4.0 ± 0.3 to 4.7 ± 0.4 mEq/L (*p* = 0.28), ionized calcium from 0.8 ± 0.1 to 0.6 ± 0.1 mmol/L (*p* = 0.38), and non-fasting glucose from 263.8 ± 36.1 to 320.8 ± 45.9 mg/dL (*p* = 0.35). Hematocrit changed from 43.4 ± 4.3% to 38.4 ± 2.2% (*p* = 0.35), whereas estimated hemoglobin, calculated from hematocrit using the formula Hb (g/dL) = Ht (%)/3, changed from 14.5 ± 1.4 to 12.8 ± 0.7 g/dL. None of these pre-MV to post-MV changes reached statistical significance.

Table 3 summarizes renal parameters in the Obese and LPS groups at the pre-MV and post-MV time points. In the Obese group, serum urea increased significantly from 40.2 ± 4.9 mg/dL at pre-MV to 53.8 ± 2.8 mg/dL at post-MV, corresponding to a mean paired increase of 13.7 mg/dL (95% CI, 8.44 to 18.9), t(4) = 7.25, *p* = 0.002, Cohen’s d = 3.24 (95% CI, 0.92 to 5.56). The urinary protein-to-creatinine ratio also increased significantly, from 21.7 ± 12.4 mg/mg at pre-MV to 31.5 ± 5.7 mg/mg at post-MV, with a mean paired increase of 9.75 mg/mg (95% CI, 0.87 to 18.8), t(4) = 3.05, *p* = 0.038, Cohen’s d = 1.36 (95% CI, 0.07 to 2.59). Serum creatinine changed from 0.9 ± 0.1 to 0.9 ± 0.07 mg/dL (*p* = 0.88), urinary creatinine from 26.2 ± 7.1 to 23.1 ± 0.7 mg/dL (*p* = 0.81), and urinary urea from 1398 ± 318 to 1140 ± 293 mg/dL (*p* = 0.62); none of these pre-MV to post-MV changes reached statistical significance.

In the LPS group, serum creatinine increased significantly from 0.5 ± 0.03 mg/dL at pre-MV to 0.7 ± 0.06 mg/dL at post-MV, corresponding to a mean paired increase of 0.241 mg/dL (95% CI, 0.021 to 0.461), t(2) = 4.71, *p* = 0.042, Cohen’s d = 2.72 (95% CI, 0.06 to 5.43). Urinary creatinine changed from 15.7 ± 4.2 to 19.4 ± 3.4 mg/dL (*p* = 0.54), serum urea from 35.7 ± 1.2 to 57.8 ± 8.1 mg/dL (*p* = 0.11), urinary urea from 1167 ± 348 to 1343 ± 391 mg/dL (*p* = 0.75), and the urinary protein-to-creatinine ratio from 4.7 ± 1.9 to 6.8 ± 2.7 mg/mg (*p* = 0.54); none of these pre-MV to post-MV changes reached statistical significance.

### 3.4. Renal Inflammatory and AT1R Protein Expression at the Post-MV Time Point

**Figure 6:**
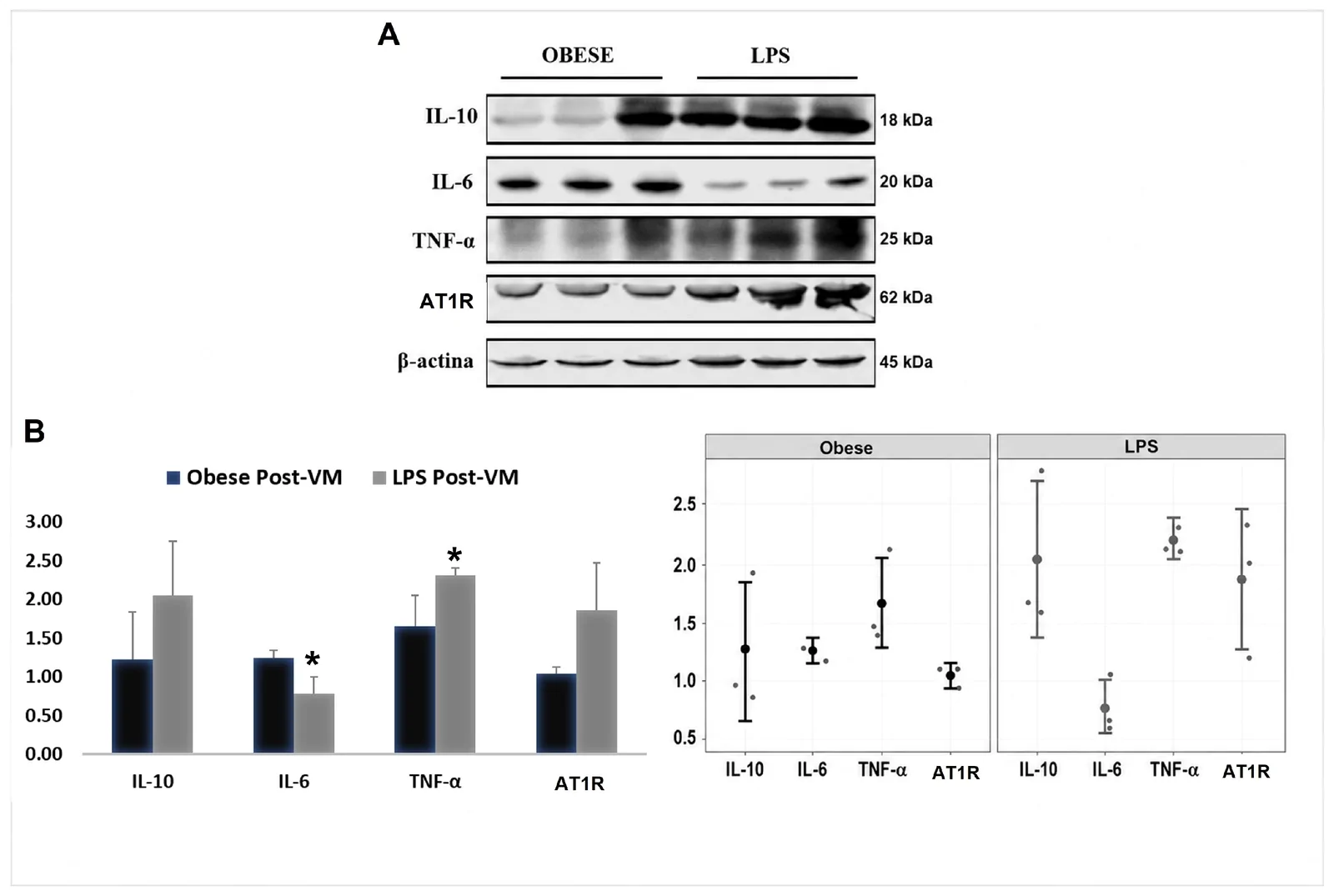
Renal protein expression at the post-MV time point in Obese and LPS rats. (**A**) Representative Western blot bands for IL-10, IL-6, TNF-α, AT1R, and β-actin. (**B**) Densitometric quantification of renal protein expression normalized to β-actin. Bar graphs show group means ± SD, and the plots on the right display individual animal data points; central symbols and error bars also represent the mean ± SD. Between-phenotype comparisons were performed at the post-MV time point (Obese, *n* = 3; LPS, *n* = 3 biological replicates). (*) *p* ≤ 0.05, Obese group versus LPS group.

Figure 6A,B, shows renal protein expression at the post-MV time point in the Obese and LPS groups. IL-10 expression was 1.23 ± 0.61 in the Obese group and 2.05 ± 0.70 in the LPS group, with no statistically significant between-group difference (*p* = 0.25). AT1R expression was 1.04 ± 0.09 in the Obese group and 1.87 ± 0.61 in the LPS group, with no statistically significant between-group difference (Mann–Whitney U = 0.00, *p* = 0.100).

IL-6 expression was significantly lower in the LPS group than in the Obese group, with a mean between-group difference (LPS − Obese) of −0.463 (95% CI, −0.828 to −0.099), t(4) = −3.53, *p* = 0.024, Cohen’s d = −2.88 (95% CI, −5.32 to −0.32). In contrast, TNF-α expression was significantly higher in the LPS group than in the Obese group, with a mean between-group difference (LPS − Obese) of 0.643 (95% CI, 0.029 to 1.26), t(4) = 2.91, *p* = 0.044, Cohen’s d = 2.37 (95% CI, 0.06 to 4.56).

## 4. Discussion

The principal contribution of this exploratory study was to describe early renal changes observed during a short-term mechanical ventilation protocol, which displayed distinct profiles across two inflammatory-metabolic contexts. These renal findings occurred in association with different systemic patterns, reinforcing the importance of interpreting the renal findings within the specific pathophysiological context of each phenotype. In this regard, acid–base, respiratory, metabolic, and hemodynamic alterations provided the physiological framework for this interpretation.

The reduction in the acidotic respiratory component was not accompanied by systemic normalization during the short-term reduced-tidal-volume mechanical ventilation protocol. In both models, the decrease in PaCO_2_ was consistent with attenuation of the respiratory component of the acid–base disturbance; however, metabolic abnormalities persisted. This finding is relevant because, under critical conditions, interpreting pH or PaCO_2_ in isolation may suggest a partial correction of acid–base balance while simultaneously obscuring the persistence of adverse metabolic and systemic alterations. The nature of these alterations, however, differed between the two experimental contexts.

In the Obese group, the significant increase in pH, accompanied by the reduction in PaCO_2_, was consistent with attenuation of the acidotic respiratory component. However, the persistence of reduced bicarbonate, negative base excess, and persistently elevated lactate was consistent with maintenance of the metabolic component of the acid–base disturbance throughout the protocol. This pattern is biologically compatible with the context of obesity; a condition associated with systemic metabolic alterations and obesity-related respiratory and acid–base disturbances [38]. The reduction in the acidotic respiratory component was not accompanied by normalization of the metabolic and systemic alterations evaluated.

In contrast, in the LPS group, PaCO_2_ decreased markedly after the protocol, reaching values consistent with pronounced hypocapnia. Although this change was associated with an increase in pH, it did not represent normalization of acid–base balance, as it occurred simultaneously with a significant decrease in bicarbonate and a significant increase in lactate. This combination of changes was consistent with persistence of the metabolic component of the acid–base disturbance in association with hypocapnia. This pattern is compatible with the context of LPS-induced endotoxemia, a model of acute systemic inflammation [13], in which alterations in microvascular perfusion, mitochondrial dysfunction, increased lactate production, and impaired lactate clearance may contribute to hyperlactatemia [39,40].

In addition to the acid–base alterations, the oxygenation data showed that the reduction in PaCO_2_ was not accompanied by a proportional improvement in gas exchange in either experimental context. Estimated PAO_2_ increased significantly in both groups, as expected from the alveolar gas equation in the setting of reduced PaCO_2_ with FiO_2_ maintained constant. However, this increase was not accompanied by a significant improvement in the PaO_2_/FiO_2_ ratio. Changes in the A–a gradient also did not reach statistical significance: the gradient decreased in the Obese group but increased in the LPS group. Since the A–a gradient increases when oxygen transfer from the alveoli to the arterial blood becomes less efficient, the increase observed in the LPS group is consistent with impaired gas exchange.

Taken together, these findings showed that the reduction in PaCO_2_ was not accompanied by a significant improvement in the gas-exchange indices evaluated. The persistence of an elevated A–a gradient despite the reduction in PaCO_2_ is physiologically more consistent with ventilation–perfusion mismatch and/or intrapulmonary shunt than with isolated alveolar hypoventilation; diffusion impairment may also have contributed [41,42,43,44]. However, because the present study did not directly assess ventilation–perfusion relationships, shunt fraction, lung recruitability, or the regional distribution of aeration, these interpretations should be considered physiologically plausible but exploratory.

Within this context of systemic and respiratory alterations, the renal findings constituted the central focus of the analysis. In the Obese group, significant increases in serum urea and proteinuria, despite stable serum creatinine, were consistent with a predominantly nitrogenous and proteinuric pattern. This combination of changes is biologically compatible with obesity as a systemic condition associated with hemodynamic, metabolic, and inflammatory alterations that may affect renal function [7].

In the LPS group, by contrast, a distinct pattern predominated, characterized by a significant increase in serum creatinine. This finding was consistent with an early increase in a renal biochemical marker in the context of acute endotoxemia, without allowing structural kidney injury to be inferred from this marker alone. This pattern is biologically plausible given the hemodynamic, microcirculatory, endothelial, and intrarenal inflammatory alterations that may impair renal perfusion, glomerular filtration, and tubular homeostasis [45,46]. Given the relatively delayed kinetics of serum creatinine, its increase should be interpreted cautiously as an early functional marker rather than as a comprehensive measure of the change in glomerular filtration over the 1 h interval.

Complementing the serum and urinary findings, renal protein expression provided additional molecular information. These analyses were performed only at the post-MV time point and represented the only direct statistical comparison between the Obese and LPS groups. Renal IL-6 expression was higher in the Obese group, whereas TNF-α expression was higher in the LPS group. These post-MV findings were consistent with distinct renal molecular profiles between the two experimental contexts. However, the study cannot determine whether the observed serum and urinary biochemical renal changes were accompanied by structural kidney injury. This interpretation is in line with the literature describing obesity as a state of chronic low-grade inflammation, often associated with increased IL-6 levels [47,48,49], and LPS exposure as being associated with TNF-α-mediated pro-inflammatory responses [50,51,52].

**Figure 7:**
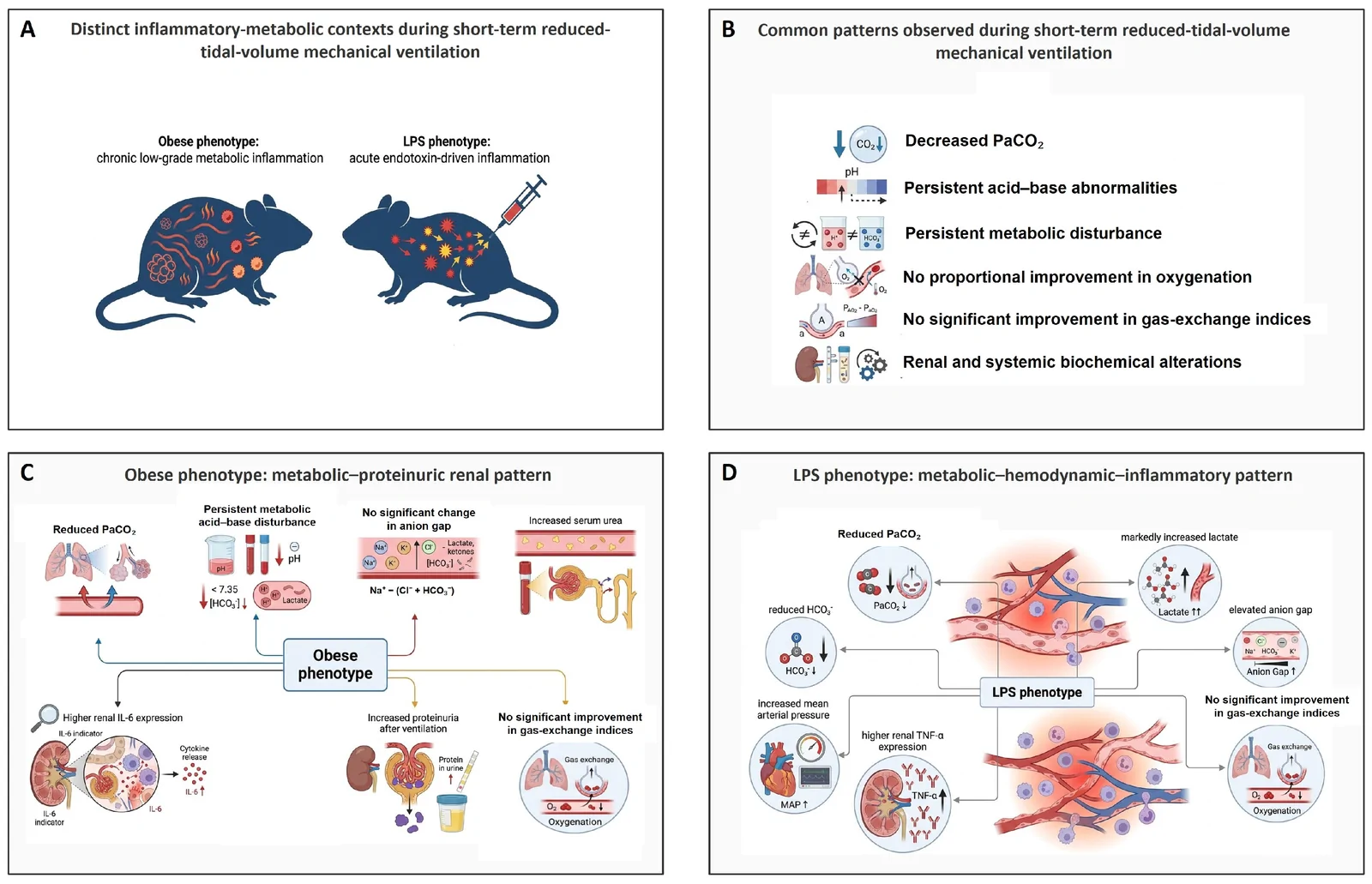
Summary of phenotype-associated systemic and renal patterns observed during short-term reduced-tidal-volume mechanical ventilation. (**A**). Diet-induced obesity and LPS-induced endotoxemia as distinct inflammatory-metabolic contexts. (**B**). Findings observed in both phenotypes, including decreased PaCO_2_, persistent metabolic acid–base abnormalities, lack of proportional improvement in oxygenation, and renal and systemic biochemical changes. (**C**). Post-MV findings in the Obese phenotype, characterized by increased serum urea, increased urinary protein-to-creatinine ratio, higher renal IL-6 expression than in the LPS group, and no significant improvement in gas-exchange indices. (**D**). Post-MV findings in the LPS phenotype, characterized by reduced HCO_3_^−^, increased lactate and mean arterial pressure, higher renal TNF-α expression than in the Obese group, and no significant improvement in gas-exchange indices. Note: mechanistic interpretations are exploratory and require confirmation in future studies.

Across both experimental contexts, common findings included a reduction in PaCO_2_, persistence of metabolic acid–base abnormalities, and no proportional improvement in the gas-exchange indices evaluated. Beyond these shared features, distinct systemic and renal patterns were observed within each experimental context. In the Obese group, the main findings were persistent metabolic alterations, increased serum urea and proteinuria, and higher renal IL-6 expression at the post-MV time point. In the LPS group, increased lactate, reduced bicarbonate, elevated mean arterial pressure, increased serum creatinine, and higher renal TNF-α expression were observed at the post-MV time point. These findings were consistent with the respective inflammatory-metabolic contexts, without establishing that these contexts were solely responsible for the observed differences and without allowing exclusive causal attribution to mechanical ventilation. Figure 7 summarizes this integrated interpretation.

The patterns observed in each phenotype were also consistent with the concept of the two-hit model [18], in which an initial insult modifies the host’s physiological state. At the same time, a second stressor may amplify or unmask subsequent systemic alterations. In this context, mechanical ventilation may have acted as an additional stressor in organisms that had been previously modified. However, the data from the present study do not allow the observed metabolic, hemodynamic, and renal alterations to be attributed specifically to mechanical ventilation.

Other experimental studies in rats have also demonstrated substantial heterogeneity in mechanical ventilation protocols, including variations in tidal volume, PEEP, respiratory rate, FiO_2_, ventilation duration, and the nature of the preceding insult [21,22,23,24,25,26,27,53]. Although tidal volumes of approximately 5–10 mL/kg are often classified as low or protective, the physiological response may depend on the combination of ventilatory parameters and the pre-existing biological context [18,53].

Experimental studies indicate that tidal volume can influence both renal and systemic responses during mechanical ventilation [54,55]. In healthy rats, high tidal-volume ventilation acutely reduced glomerular filtration [54], whereas in septic or endotoxin-exposed animals, greater ventilatory intensity was associated with renal apoptosis, renal hypoperfusion, endothelial dysfunction, increased NGAL expression, and impaired renal function [46,56,57]. These effects also varied according to the underlying pathological condition, suggesting that ventilatory exposure and host pathophysiology may interact [56,58].

This observation is consistent with the findings of the present study, in which a reduced-tidal-volume strategy was associated with different systemic and renal patterns in each phenotype, without significant improvement in the gas-exchange indices evaluated. Therefore, the difference in body-weight-normalized tidal volume between the Obese and LPS groups may also have contributed to the distinct systemic and renal patterns observed and should be considered when interpreting phenotype-associated responses.

This perspective underscores the difficulty of disentangling, at both the molecular and functional levels, biotrauma from the host inflammatory context, which remains one of the major challenges in contemporary critical care medicine [59,60]. In clinical practice, biological responses potentially related to the mechanical forces generated by the ventilator may overlap with pathways already activated by the underlying disease, comorbidities, and the systemic immune response, thereby complicating identification of the specific contribution of ventilatory support to the observed alterations. In the experimental setting, even the inclusion of a ventilated control group may not eliminate the influence of the pre-existing biological state, because healthy animals and animals previously exposed to an initial insult may respond differently to the same ventilatory stressor. From this perspective, the two-hit model remains relevant for interpreting the potential interplay between the host’s baseline condition and mechanical ventilation.

It is important to emphasize that all pre- to post-MV changes were observed during a short-term integrated experimental protocol that included anesthesia, surgical instrumentation, and mechanical ventilation. Therefore, the study design does not allow the independent contribution of each component to be isolated, and the observed physiological, metabolic, hemodynamic, and renal alterations should not be attributed exclusively to mechanical ventilation. Accordingly, the findings do not support a definitive causal attribution. However, they support the hypothesis that the response to ventilatory support may be influenced not only by tidal volume reduction but also by the host’s underlying pathophysiological and inflammatory-metabolic context.

### Study Limitations

This study has several limitations. The exploratory design included a small sample size and limited statistical power to detect small-to-moderate effects, requiring caution in the interpretation of non-significant findings. In addition, mechanical ventilation was applied for only 1 h, allowing the assessment of early responses but not of later metabolic, renal, or inflammatory trajectories. Renal assessment was primarily based on serum and urinary biochemical parameters, and specific markers of tubular injury, such as neutrophil gelatinase-associated lipocalin (NGAL) and kidney injury molecule-1 (KIM-1), as well as renal histological evaluation, were not performed. Therefore, the present study cannot determine whether the observed serum and urinary biochemical renal changes were accompanied by structural kidney injury. Renal protein expression was assessed only at the post-MV time point because kidney tissue collection required euthanasia; therefore, baseline renal protein expression could not be obtained from the same animals, precluding assessment of temporal changes in these molecular markers. Estimated alveolar oxygen pressure (PAO_2_) and the alveolar–arterial oxygen gradient were calculated assuming a respiratory quotient of 0.8, without direct measurement by indirect calorimetry. Plateau pressure, intrinsic PEEP, expired tidal volume, driving pressure, and respiratory system compliance were not measured; consequently, the mechanical load applied to the respiratory system and the equivalence of pulmonary stress between groups could not be fully characterized. Because anesthesia, surgical instrumentation, and mechanical ventilation were components of the same experimental protocol, the independent contribution of each component to the observed changes could not be isolated. Finally, a combined Obese + LPS group was initially considered but could not be completed because of mortality observed during the experimental protocol. Consequently, this group was not included in the statistical analyses or in the main results, limiting the direct evaluation of the potential interaction between obesity and endotoxemia.

## 5. Conclusions

The present study underscores the challenges involved in interpreting systemic responses to mechanical ventilation in clinical practice. In this exploratory study, attenuation of the respiratory component of acidosis was observed in both inflammatory contexts in association with an integrated protocol of anesthesia, surgical instrumentation, and short-term reduced-tidal-volume mechanical ventilation, without proportional normalization of the systemic alterations. In the Obese group, the main findings were persistence of the metabolic disturbance, increased serum urea and proteinuria, and higher renal IL-6 expression at the post-protocol time point. In the LPS group, increased lactate, reduced bicarbonate, elevated mean arterial pressure, increased serum creatinine, and higher renal TNF-α expression were observed at the post-protocol time point. The observed systemic and renal findings are consistent with an association with the underlying pathophysiology and inflammatory-metabolic state of the host. In addition, the data generate hypotheses for future studies directly comparing standardized ventilatory strategies with strategies adapted to the pre-existing phenotype.

## Figures and Tables

**Table 1:** Arterial blood gas parameters and mean arterial pressure in the Obese and LPS groups at the pre-MV and post-MV time points. PaCO_2_, arterial partial pressure of carbon dioxide; PaO_2_, arterial partial pressure of oxygen; HCO_3_^−^, bicarbonate; BE-B, blood base excess; MAP, mean arterial pressure. Values are presented as mean ± SEM. (*) *p* ≤ 0.05 versus the respective pre-MV period within the same group.

Arterial Blood Gas	Obese Group		LPS Group	
Physiological reference	Pre-MV	Post-MV	Pre-MV	Post-MV
pH (7.29–7.38)	7.1 ± 0.06	7.2 ± 0.02 *	7.1 ± 0.1	7.2 ± 0.08
PaCO_2_ (40–53 mmHg)	51.9 ± 6.3	31.3 ± 2.1 *	46.6 ± 10.3	21.4 ± 3.5 *
PaO_2_ (87–106 mmHg)	110.6 ± 36.2	132.2 ± 33.5	80.2 ± 14.4	90.9 ± 14.6
HCO_3_^−^ (22–26 mEq/L)	16.3 ± 1.6	14.1 ± 1.2	19.6 ± 0.7	12.0 ± 1.9 *
Chloride (98–110 mmol/L)	116.8 ± 1.9	117.2 ± 2.1	116.0 ± 3.2	113.0 ± 1.3
BE-B (−4 to +2 mmol/L)	−10.8 ± 4.1	−11.3 ± 3.1	−8.9 ± 7.1	−5.6 ± 5.4
Lactate (0.5–1.5 mmol/L)	2.7 ± 1.0	3.2 ± 0.6	1.5 ± 0.2	4.8 ± 1.2 *
MAP (80–120 mmHg)	97.0 ± 9.9	102.2 ± 9.6	119.5 ± 6.3	148.5 ± 11.4 *

Note: Values in parentheses indicate published physiological ranges for Wistar rats [34], provided only as biological context. These values were not obtained from contemporaneous healthy control animals, were not included in the statistical analysis, and should not be interpreted as a formal control group.

**Table 2:** Biochemical parameters in the Obese and LPS groups at the pre-MV and post-MV time points. Na^+^, sodium; K^+^, potassium; Ca^2+^, ionized calcium; non-fasting glucose, blood glucose measured without fasting; Ht, hematocrit; Hb, hemoglobin. Values are presented as mean ± SEM.

Biochemical Parameters	Obese Group		LPS Group	
Physiological reference	Pre-MV	Post-MV	Pre-MV	Post-MV
Na^+^, sodium (142–151 mEq/L)	152.0 ± 2.7	153.5 ± 3.1	145.7 ± 3.1	145.7 ± 5.5
K^+^, potassium (3.8–5.5 mEq/L)	4.2 ± 0.2	4.2 ± 0.2	4.0 ± 0.3	4.7 ± 0.4
Ca^2+^ (1.11–1.14 mmol/L)	0.5 ± 0.05	0.4 ± 0.06	0.8 ± 0.1	0.6 ± 0.1
Non-fasting glucose (70–208 mg/dL)	276.0 ± 30.7	325.4 ± 32.4	263.8 ± 36.1	320.8 ± 45.9
Hematocrit (39–52%)	35.4 ± 1.8	34.4 ± 2.0	43.4 ± 4.3	38.4 ± 2.2
Hemoglobin (13–17 g/dL)	11.8 ± 0.6	11.5 ± 0.7	14.5 ± 1.4	12.8 ± 0.7

Note: Values in parentheses indicate published physiological ranges for Wistar rats [35], provided only as biological context. These values were not obtained from contemporaneous healthy control animals, were not included in the statistical analysis, and should not be interpreted as a formal control group.

**Table 3:** Renal parameters in the Obese and LPS groups at the pre-MV and post-MV time points. Values are presented as mean ± SEM. (*) *p* ≤ 0.05 versus the respective pre-MV period within the same group.

Renal Parameters	Obese Group		LPS Group	
Physiological reference	Pre-MV	Post-MV	Pre-MV	Post-MV
Serum creatinine (0.30–0.60 mg/dL)	0.9 ± 0.1	0.9 ± 0.07	0.5 ± 0.03	0.7 ± 0.06 *
Urinary creatinine (8–25 mg/dL)	26.2 ± 7.1	23.1 ± 0.7	15.7 ± 4.2	19.4 ± 3.4
Serum urea (20–50 mg/dL)	40.2 ± 4.9	53.8 ± 2.8 *	35.7 ± 1.2	57.8 ± 8.1
Urinary urea (300–600 mg/dL)	1398 ± 318	1140 ± 293	1167 ± 348	1343 ± 391
Urinary protein-to-creatinine ratio mg/mg (<7 mg/mg)	21.7 ± 12.4	31.5 ± 5.7 *	4.7 ± 1.9	6.8 ± 2.7

Note: Values in parentheses indicate published physiological ranges for Wistar rats [36,37], provided only as biological context. These values were not obtained from contemporaneous healthy control animals, were not included in the statistical analysis, and should not be interpreted as a formal control group.

## Data Availability

The data supporting the reported results are available on request from the corresponding author.

## References

[1] Merola R., Vargas M., Battaglini D. (2025). Ventilator-Induced Lung Injury: The Unseen Challenge in Acute Respiratory Distress Syndrome Management. J. Clin. Med..

[2] Yang H., Benos P.V., Kitsios G.D. (2020). Protecting the Lungs but Hurting the Kidneys: Causal Inference Study for the Risk of Ventilation-Induced Kidney Injury in ARDS. Ann. Transl. Med..

[3] Benites M.H., Suarez-Sipmann F., Kattan E., Cruces P., Retamal J. (2025). Ventilation-Induced Acute Kidney Injury in Acute Respiratory Failure: Do PEEP Levels Matter?. Crit. Care.

[4] Muñoz J., Cedeño J.A., Castañeda G.F., Visedo L.C. (2024). Personalized Ventilation Adjustment in ARDS: A Systematic Review and Meta-Analysis of Image, Driving Pressure, Transpulmonary Pressure, and Mechanical Power. Heart Lung.

[5] Merola R., Battaglini D., Schultz M.J., Rocco P.R.M. (2026). Physiology-Guided Personalized Mechanical Ventilation to Prevent Ventilator-Induced Lung Injury. Front. Med..

[6] Leite T.T., Gomes C.A.M., Valdivia J.M.C., Libório A.B. (2019). Respiratory Parameters and Acute Kidney Injury in Acute Respiratory Distress Syndrome: A Causal Inference Study. Ann. Transl. Med..

[7] Pessoa E.A., Convento M.B., Castino B., Leme A.M., de Oliveira A.S., Aragão A., Fernandes S.M., Carbonel A., Dezoti C., Vattimo M.F. (2020). Beneficial Effects of Isoflavones in the Kidney of Obese Rats Are Mediated by PPAR-Gamma Expression. Nutrients.

[8] Athayde R.A.B., Oliveira Filho J.R.B., Lorenzi Filho G., Genta P.R. (2018). Obesity Hypoventilation Syndrome: A Current Review. J. Bras. Pneumol..

[9] Tafelski S., Yi H., Ismaeel F., Krannich A., Spies C., Nachtigall I. (2016). Obesity in Critically Ill Patients Is Associated with Increased Need of Mechanical Ventilation but Not with Mortality. J. Infect. Public Health.

[10] Wang Y., Li D., Zhu L. (2026). Obesity Paradox in Critically Ill Patients: Do Patients with Critical Diseases Benefit from Obesity?. Obes. Facts.

[11] Rabec C., Janssens J.P., Murphy P.B. (2025). Ventilation in the Obese: Physiological Insights and Management. Eur. Respir. Rev..

[12] Lambert D.C., Kane J., Slaton A., Abramowitz M.K. (2022). Associations of Metabolic Syndrome and Abdominal Obesity with Anion Gap Metabolic Acidosis among US Adults. Kidney360.

[13] Anhê F.F., Barra N.G., Cavallari J.F., Henriksbo B.D., Schertzer J.D. (2021). Metabolic Endotoxemia Is Dictated by the Type of Lipopolysaccharide. Cell Rep..

[14] Kriswidyatomo P., Pradnyan Kloping Y., Guntur Jaya M., Adrian Nugraha R., Prawira Putri C., Hendrawan Putra D., Ananda Kloping N., Adityawardhana T., Yogiswara N., Margarita Rehatta N. (2022). Prognostic Value of PCO_2_ Gap in Adult Septic Shock Patients: A Systematic Review and Meta-Analysis. Turk. J. Anaesthesiol. Reanim..

[15] Shen X., He L., Cai W. (2024). Role of Lipopolysaccharides in the Inflammation and Pyroptosis of Alveolar Epithelial Cells in Acute Lung Injury and Acute Respiratory Distress Syndrome. J. Inflamm. Res..

[16] Kuipers M.T., van der Poll T., Schultz M.J., Wieland C.W. (2011). Bench-to-Bedside Review: Damage-Associated Molecular Patterns in the Onset of Ventilator-Induced Lung Injury. Crit. Care.

[17] Curley G.F., Laffey J.G., Zhang H., Slutsky A.S. (2016). Biotrauma and Ventilator-Induced Lung Injury: Clinical Implications. Chest.

[18] Bilodeaux J., Farooqi H., Osovskaya M., Sosa A., Wallbank A., Knudsen L., Sottile P.D., Albers D.J., Smith B.J. (2023). Differential Effects of Two-Hit Models of Acute and Ventilator-Induced Lung Injury on Lung Structure, Function, and Inflammation. Front. Physiol..

[19] Marques R.G., Morales M.M., Petroianu A. (2009). Brazilian Law for Scientific Use of Animals. Acta Cir. Bras..

[20] Patel S., Dhande I., Gray E.A., Ali Q., Hussain T. (2019). Prevention of lipopolysaccharide-induced CD11b+ immune cell infiltration in the kidney: Role of AT2 receptors. Biosci. Rep..

[21] Ball L., Pelosi P. (2019). How I Ventilate an Obese Patient. Crit. Care.

[22] De Jong A., Wrigge H., Hedenstierna G., Gattinoni L., Chiumello D., Frat J.P., Ball L., Schetz M., Pickkers P., Jaber S. (2020). How to Ventilate Obese Patients in the ICU. Intensive Care Med..

[23] De Jong A., Chanques G., Jaber S. (2017). Mechanical Ventilation in Obese ICU Patients: From Intubation to Extubation. Crit. Care.

[24] Jones R., Gittens J., Manuel A., Esquinas A., Lemyze M. (2018). Ventilation Modes for Obese Patients Under Mechanical Ventilation. Mechanical Ventilation in the Critically Ill Obese Patient.

[25] Gorman E.A., O’Kane C.M., McAuley D.F. (2022). Acute Respiratory Distress Syndrome in Adults: Diagnosis, Outcomes, Long-Term Sequelae, and Management. Lancet.

[26] Wang X., Bai C., Chen H. (2010). The Value of the Lipopolysaccharide-Induced Acute Lung Injury Model in Respiratory Medicine. Expert Rev. Respir. Med..

[27] Fonseca L.M.C., Reboredo M.M., Lucinda L.M.F., Fazza T.F., Bergamini B.C., Botelho M.P., Lopes G.M., Ferreira J.N.D., Carvalho E.V., Pinheiro B.V. (2023). Effects of Atelectatic Areas on the Surrounding Lung Tissue During Mechanical Ventilation in an Experimental Model of Acute Lung Injury Induced by Lipopolysaccharide. Crit. Care Sci..

[28] Zhou H., Fu Z., Wei Y., Liu J., Cui X., Yang W., Ding W., Pan P., Li W. (2013). Hydrogen Inhalation Decreases Lung Graft Injury in Brain-Dead Donor Rats. J. Heart Lung Transplant..

[29] Peralta-Ramírez A., Raya A.I., Pineda C., Rodríguez M., Aguilera-Tejero E., López I. (2014). Acid-Base Balance in Uremic Rats with Vascular Calcification. Nephron Extra.

[30] Rinaldi S., De Gaudio A.R. (2005). Strong ion difference and strong anion gap: The Stewart approach to acid base disturbances. Curr. Anaesth. Crit. Care.

[31] Hendrix J.M., Burns B. (2026). Alveolar Gas Equation. StatPearls.

[32] Taussky H.H. (1956). A Procedure Increasing the Specificity of the Jaffe Reaction for the Determination of Creatine and Creatinine in Urine and Plasma. Clin. Chim. Acta.

[33] Lowry O.H., Rosebrough N.J., Farr A.L., Randall R.J. (1951). Protein Measurement with the Folin Phenol Reagent. J. Biol. Chem..

[34] Subramanian R.K., Sidharthan A., Maneksh D., Ramalingam L., Manickam S.A., Kanthakumar P., Subramani S. (2013). Normative Data for Arterial Blood Gas and Electrolytes in Anesthetized Rats. Indian J. Pharmacol..

[35] Giknis M.L.A., Clifford C.B. (2008). Clinical Laboratory Parameters for Crl:WI(Han).

[36] Harkness J.E., Wagner J.E. (1993). Biologia e Clínica de Coelhos e Roedores.

[37] Castro B.B.A., Colugnati F.A.B., Cenedeze M.A., Suassuna P.G.A., Pinheiro H.S. (2014). Padronização da Avaliação da Função Renal de Ratos (*Rattus norvegicus*) Wistar do Biotério da Universidade Federal de Juiz de Fora. Braz. J. Nephrol..

[38] Mokhlesi B., Masa J.F., Brozek J.L., Gurubhagavatula I., Murphy P.B., Piper A.J., Tulaimat A., Afshar M., Balachandran J.S., Dweik R.A. (2019). Evaluation and Management of Obesity Hypoventilation Syndrome. An Official American Thoracic Society Clinical Practice Guideline. Am. J. Respir. Crit. Care Med..

[39] Sun Z., Song Y., Li J., Li Y., Yu Y., Wang X. (2023). Potential Biomarker for Diagnosis and Therapy of Sepsis: Lactylation. Immun. Inflamm. Dis..

[40] Imig J.D., Ryan M.J. (2013). Immune and Inflammatory Role in Renal Disease. Compr. Physiol..

[41] Hantzidiamantis P.J., Amaro E. (2026). Physiology, Alveolar to Arterial Oxygen Gradient. StatPearls.

[42] Nagato A.C., Machado-Junior P.A., Valenca S.S., Russo R.C., Bezerra F.S. (2026). Experimental Models of Acute Lung Injury to Study Inflammation and Pathophysiology: A Narrative Review. Antioxidants.

[43] Banerjee D., Yee B.J., Piper A.J., Zwillich C.W., Grunstein R.R. (2007). Obesity hypoventilation syndrome: Hypoxemia during continuous positive airway pressure. Chest.

[44] Piagnerelli M., Boudjeltia K.Z., Vanhaeverbeek M., Vincent J.L. (2003). Red Blood Cell Rheology in Sepsis. Intensive Care Med..

[45] Nguyen A.T., Mandard S., Dray C., Deckert V., Valet P., Besnard P., Drucker D.J., Lagrost L., Grober J. (2014). Lipopolysaccharides-Mediated Increase in Glucose-Stimulated Insulin Secretion: Involvement of the GLP-1 Pathway. Diabetes.

[46] Vaschetto R., Kuiper J.W., Musters R.J., Eringa E.C., Della Corte F., Murthy K., Groeneveld A.B., Plötz F.B. (2010). Renal Hypoperfusion and Impaired Endothelium-Dependent Vasodilation in an Animal Model of VILI: The Role of the Peroxynitrite-PARP Pathway. Crit. Care.

[47] Saad K.M., Gad M.S., Tang J., Capehart K., Abdelsayed R., Williams J.M., Elmarakby A. (2025). Obesity Promotes Renal Inflammation and Fibrosis Independent of Sex in SS Leptin Receptor Mutant (SS^LepR^) Rats. Biomedicines.

[48] Stemmer K., Perez-Tilve D., Ananthakrishnan G., Bort A., Seeley R.J., Tschöp M.H., Dietrich D.R., Pfluger P.T. (2012). High-Fat-Diet-Induced Obesity Causes an Inflammatory and Tumor-Promoting Microenvironment in the Rat Kidney. Dis. Model. Mech..

[49] Zhang L., Xu F., Hou L. (2024). IL-6 and Diabetic Kidney Disease. Front. Immunol..

[50] Shin S.H., Kim E.K., Lee K.Y., Kim H.S. (2019). TNF-α Antagonist Attenuates Systemic Lipopolysaccharide-Induced Brain White Matter Injury in Neonatal Rats. BMC Neurosci..

[51] Noiri E., Kuwata S., Nosaka K., Tokunaga K., Juji T., Shibata Y., Kurokawa K. (1994). Tumor Necrosis Factor-Alpha mRNA Expression in Lipopolysaccharide-Stimulated Rat Kidney. Chronological Analysis of Localization. Am. J. Pathol..

[52] Eliyatkın N.Ö., İşlek A., Durmaz S., Ayyıldız F., Rahman Ö. (2024). Can Adalimumab Prevent from Acute Effects of Lipopolysaccharide Induced Renal Injury in Rats?. Acta Cir. Bras..

[53] Joelsson J.P., Karason S. (2025). Ventilator-Induced Lung Injury in Rat Models: Are They All Equal in the Race?. Lab. Anim. Res..

[54] Luque A., Shimizu M.H.M., Andrade L., Sanches T.R., Seguro A.C. (2009). Glomerular Filtration Is Reduced by High Tidal Volume Ventilation in an In Vivo Healthy Rat Model. Braz. J. Med. Biol. Res..

[55] Haitsma J.J., Uhlig S., Verbrugge S.J., Göggel R., Poelma D.L.H., Lachmann B. (2003). Injurious Ventilation Strategies Cause Systemic Release of IL-6 and MIP-2 in Rats In Vivo. Clin. Physiol. Funct. Imaging.

[56] Kuiper J.W., Groeneveld A.B.J., Haitsma J.J., Smeding L., Begieneman M.P.V., Jothy S., Vaschetto R., Plötz F.B. (2014). Injurious Mechanical Ventilation Causes Kidney Apoptosis and Dysfunction during Sepsis but Not after Intra-Tracheal Acid Instillation: An Experimental Study. BMC Nephrol..

[57] Vaschetto R., Kuiper J.W., Chiang S.R., Haitsma J.J., Juco J.W., Uhlig S., Plötz F.B., Della Corte F., Zhang H., Slutsky A.S. (2008). Inhibition of Poly(adenosine Diphosphate-Ribose) Polymerase Attenuates Ventilator-Induced Lung Injury. Anesthesiology.

[58] Crimi E., Zhang H., Han R.N.N., Del Sorbo L., Ranieri V.M., Slutsky A.S. (2006). Ischemia and Reperfusion Increases Susceptibility to Ventilator-Induced Lung Injury in Rats. Am. J. Respir. Crit. Care Med..

[59] Costa A., Sakho B., Gomez S., Khanyan B., Leybengrub P., Bergese S. (2025). Ventilator-Associated Lung Injury: Pathophysiology, Prevention, and Emerging Therapeutic Strategies. Int. J. Mol. Sci..

[60] Li L., Yang D., Yang M., Zhang H., Li L., Lu X., Wang L., Luo H. (2026). Ventilator-Induced Lung Injury: From Mechanisms to Integrated Clinical Management. Front. Med..

